# The Influence of Domain Permutations of an Albumin-Binding Domain-Fused HER2-Targeting Affibody-Based Drug Conjugate on Tumor Cell Proliferation and Therapy Efficacy

**DOI:** 10.3390/pharmaceutics13111974

**Published:** 2021-11-21

**Authors:** Wen Yin, Tianqi Xu, Mohamed Altai, Maryam Oroujeni, Jie Zhang, Anzhelika Vorobyeva, Olga Vorontsova, Sergey V. Vtorushin, Vladimir Tolmachev, Torbjörn Gräslund, Anna Orlova

**Affiliations:** 1 Department of Protein Science, KTH Royal Institute of Technology, 100 44 Stockholm, Sweden; wenyin@kth.se (W.Y.); jiezha@kth.se (J.Z.); 2 Department of Immunology, Genetics and Pathology, Uppsala University, 752 37 Uppsala, Sweden; Tianqi.xu@igp.uu.se (T.X.); mohamed.altai@med.lu.se (M.A.); maryam.oroujeni@igp.uu.se (M.O.); anzhelika.vorobyeva@igp.uu.se (A.V.); olga.vorontsova@igp.uu.se (O.V.); Vladimir.tolmachev@igp.uu.se (V.T.); 3 Division of Oncology, Department of Clinical Sciences Lund, Lund University, 221 84 Lund, Sweden; 4 Pathology Department, Siberian State Medical University, 634050 Tomsk, Russia; vtorushin.sv@ssmu.ru; 5 General and Molecular Pathology Department, Cancer Research Institute, Tomsk National Research Medical Center, Russian Academy of Sciences, 634009 Tomsk, Russia; 6 Research Centrum for Oncotheranostics, Research School of Chemistry and Applied Biomedical Sciences, Tomsk Polytechnic University, 634050 Tomsk, Russia; 7 Department of Medicinal Chemistry, Uppsala University, 751 23 Uppsala, Sweden; 8 Science for Life Laboratory, Uppsala University, 752 37 Uppsala, Sweden

**Keywords:** HER2, affibody molecule, albumin-binding domain, drug conjugate, targeted therapy, mertansine, DM1

## Abstract

Human epidermal growth factor receptor 2 (HER2) is a clinically validated target for breast cancer therapy. Previously, a drug-fused HER2-targeting affinity protein construct successfully extended the survival of mice bearing HER2-expressing xenografts. The aim of this study was to evaluate the influence of the number and positioning of the protein domains in the drug conjugate. Seven HER2-targeting affibody-based constructs, including one or two affibody molecules (Z) with or without an albumin-binding domain (ABD), namely Z, Z-ABD, ABD-Z, Z-Z, Z-Z-ABD, Z-ABD-Z, and ABD-Z-Z, were evaluated on their effects on cell growth, in vivo targeting, and biodistribution. The biodistribution study demonstrated that the monomeric constructs had longer blood retention and lower hepatic uptake than the dimeric ones. A dimeric construct, specifically ABD-Z-Z, could stimulate the proliferation of HER2 expressing SKOV-3 cells in vitro and the growth of tumors in vivo, whereas the monomeric construct Z-ABD could not. These two constructs demonstrated a therapeutic effect when coupled to mcDM1; however, the effect was more pronounced for the non-stimulating Z-ABD. The median survival of the mice treated with Z-ABD-mcDM1 was 63 days compared to the 37 days for those treated with ABD-Z-Z-mcDM1 or for the control animals. Domain permutation of an ABD-fused HER2-targeting affibody-based drug conjugate significantly influences tumor cell proliferation and therapy efficacy. The monomeric conjugate Z-ABD is the most promising format for targeted delivery of the cytotoxic drug DM1.

## 1. Introduction

Breast cancer is the most frequent cancer type among women. Between 15% and 30% of breast cancer tumors overexpress human epidermal growth factor receptor 2 (HER2). They are known as HER2-positive breast cancers [1] and, for these patients, there is a poorer prognosis with an increased risk of disease recurrence [2]. Monoclonal antibody (mAb) drugs targeting HER2, such as trastuzumab [3] and pertuzumab [4], are effective for some HER2-positive breast cancer patients. Two HER2-targeting antibody drug conjugates (ADCs), namely trastuzumab emtansine and trastuzumab deruxtecan, are also used clinically and demonstrated their efficacy for some HER2-positive patients. However, many patients eventually progress, and additional treatment modalities are needed.

Inspired by the specificity of antibodies and their derivatives, engineered alternative scaffold proteins have been generated to bind selectively to HER2. Affibody molecules [5], DARPins [6], ADAPTs [7], and fynomers [8] are four representative engineered alternative scaffold proteins that have been shown functional for targeting HER2-positive tumors in animal models [9]. Furthermore, affibody molecules, ADAPTs, and DARPins have been used successfully to target HER2-positive tumors in breast cancer patients for radionuclide molecular imaging of HER2 status in Phase I clinical trials [10,11,12].

Similar to the other ErbB family members, HER2 functions by forming homodimers, heterodimers, or possibly higher-order oligomers with itself or other proteins in the same family [13]. Upon dimerization, the intracellular part of the receptors will cross-phosphorylate each other to activate several intracellular signaling pathways, including the mitogen-activated protein kinase (MAPK) pathway, the signal transducer and activator of transcription (STAT) pathway, protein kinase C (PKC), and the phosphoinositide 3-kinase (PI3K/Akt) pathway [14]. These signaling pathways play roles in tumorigenesis and are involved in tumor cell proliferation as well as apoptosis [15]. Affinity proteins interacting with HER2 may affect HER2’s triggering of these pathways and thereby affect cell proliferation as well as apoptosis. For instance, trastuzumab has been shown to inhibit the growth of some HER2-positive cells [16]. Furthermore, different homo and heterodimerized DARPins and affibody dimers have been shown to either inhibit or promote the growth of HER2-positive cells [17,18].

Affibody molecules are small engineered alternative scaffold proteins based on a three-helix bundle domain framework. They are derived from the Z domain originating from staphylococcal protein A. From affibody libraries, binders with high affinity to desired targets have been selected [19]. For example, an optimized binder to HER2 (Z_HER2:2891_) has been generated with an equilibrium dissociation constant (K_D_) of 66 pM [20]. Compared to full-length antibodies, the affibody molecule is more than 20 times smaller (6.5 vs. 150 kDa). A smaller size is beneficial for penetration into solid tumors [21,22], which should lead to a more efficient distribution in the tumor mass and thus more efficient treatment. In addition, many ADCs under clinical development and approved by the US Food and Drug Administration use non-specific conjugation chemistries of the drugs, which leads to a mixture of molecules with different drug to antibody ratios (DAR). Species with different DAR may behave differently in terms of, e.g., binding specificity and in vivo stability [23,24], with high-DAR molecules being more prone to unspecific uptake in the liver, potentially leading to liver damage. By contrast, the affibody scaffold is devoid of cysteine amino acids and one or more cysteines can be inserted at desired positions to be used for site-specific conjugation of the desired number of drug molecules [25,26]. We have previously shown that it is a straightforward process to manufacture homogenous drug conjugates based on affibody molecules with a desired drug-to-affibody ratio [26].

Affibody molecules have a short plasma half-life due to their small size. It is beneficial for imaging purposes because the unbound tracer is quickly cleared from circulation. However, for therapy applications, a long plasma half-life is usually desirable. One method to prolong the plasma half-life of small molecules, including affibody molecules, is to attach them to an albumin-binding domain (ABD) [27]. A particularly useful ABD is the 46 amino acid G148-GA3 domain, derived from streptococcal protein G, and its engineered version ABD_035_, which has strong femtomolar affinity for human serum albumin (HSA) [28]. The ABD may extend the serum half-life by forming a complex with serum albumin (SA) in the blood to enlarge the complex to prevent kidney filtration. In addition, SA naturally has a long plasma half-life due to its interaction with the intracellular neonatal Fc receptor (FcRn), which rescues SA from lysosomal degradation by cells in contact with blood [28,29]. Forming a complex with SA therefore allows the fusion protein to piggy-back on the rescue mechanism, further increasing the plasma half-life of the fusion protein. The ABD_035_ also recognizes SA from other species but with a lower affinity: mouse serum albumin (MSA), with a nanomolar affinity, and bovine serum albumin (BSA), with a micromolar affinity [30]. 

Both affibody molecules and ABDs appear to be well-tolerated and safe for use in humans both in low-dose single injections for radionuclide molecular imaging [10] as well as in higher-dose multiple injection applications. For example, an ABD-containing anti-IL-17A affibody construct demonstrated safety and tolerability in a Phase 2 clinical study, which exceeded two years and included high-dose administration to patients with moderate-to-severe psoriasis (NCT03591887). 

Previously, we created a dimeric anti-HER2 ABD-fused conjugate (Z_HER2:2891_)_2_-ABD with a maleimidocaproyl linker (mc) for targeted delivery of the mertansine-derivate DM1. We found that (Z_HER2:2891_)_2_-ABD-mcDM1 effectively extended the survival of mice bearing HER2-positive SKOV-3 ovarian cancer-derived tumors from 45 days to 70 days [25]. However, in that study, the non-toxic control (Z_HER2:2891_)_2_-ABD, lacking the cytotoxic DM1, decreased the average survival due to promoted tumor growth and a higher incidence of metastasis. Ekerljung and co-workers [31] studied an earlier version of (Z_HER2:2891_)_2_ with weaker affinity, namely (Z_HER2:4_)_2_, and found that it promotes SKOV-3 cell proliferation, which corroborates the observation of increased tumor growth in our study. Therefore, we hypothesized that an anti-HER2 affibody-based dimeric drug conjugate would also affect cell proliferation. Proliferation stimulation could either increase DM1 efficacy, since DM1 is a microtubule polymerization inhibitor with high activity against cells with a high mitotic rate [32], or decrease its efficacy since the tumors would proliferate fast and thus require more drug conjugate for an efficient therapeutic effect.

In this study, we designed seven Z_HER2:2891_-based mono and dimers with or without an ABD (further denoted as Z, Z-ABD, ABD-Z, ZZ, ZZ-ABD, Z-ABD-Z, and ABD-ZZ, where Z denotes Z_HER2:2891_) and evaluated their biochemical properties, effects on cell proliferation, and biodistribution pattern. Subsequently, one of the monomeric variants, namely Z-ABD, which had a favorable biodistribution and did not promote SKOV-3 cell proliferation, and a dimeric variant, namely ABD-ZZ, which also had a favorable biodistribution but promoted SKOV-3 cell proliferation, were chosen for further analysis. Both constructs were derivatized with mcDM1 and their therapeutic properties were evaluated in a pre-clinical murine model of ovarian cancer.

## 2. Materials and Methods

All chemicals and kits were from Thermo Fisher Scientific (Waltham, MA, USA) unless indicated otherwise in the descriptions. All cell lines were from ATCC through LGC Promochem (Borås, Sweden) and were cultivated according to ATCC’s recommendations. 

The animal experiments were planned and performed in accordance with national legislation on laboratory animals’ protection. The animal studies were approved by the local authorities for laboratory animal welfare, the Ethics Committee for Animal Research in Uppsala, (approval number C86/15, 26 August 2016). 

The data were analyzed by unpaired two-tailed *t*-tests for the comparison of two values and one-way ANOVA with Bonferroni correction for the comparison of multiple values. *T*-tests, ANOVAs, Kaplan–Meier survival, Pearson’s chi-square tests, log-rank (Mantel–Cox) tests, and log-rank tests for trends were analyzed using GraphPad Prism (version 9.0.2 for Windows, GraphPad Software, La Jolla, CA, USA) to determine significant statistical differences (*p* < 0.05).

### 2.1. Construction of Genes Encoding the Affibody Constructs

The affibody used in this study was Z_HER2:2891_, specifically binding to HER2, with an equilibrium dissociation constant (K_D_) of 66 pM [20]. It is hereafter denoted as Z. The albumin-binding domain was ABD_035_ [33] and is hereafter denoted as ABD. The pET21a(+) plasmid (Merck, Darmstadt, Germany) was used as the vector for building genes encoding the constructs: Z, Z-ABD, ABD-Z, Z-Z, Z-Z-ABD, Z-ABD-Z, and ABD-Z-Z. Nucleotides encoding the amino acid sequence Gly-Gly-Gly-Gly-Ser were inserted as a linker between each affibody domain and the ABD. Nucleotides encoding a Met-His-Glu-His-Glu-His-Glu-tag were added to the N-terminus of the constructs for subsequent purification and radioisotope labeling. All genes were synthesized by Invitrogen (Waltham, MA, USA). After considering the results of the in vitro proliferation test and the in vivo biodistribution results, Z-ABD-mcDM1 and ABD-Z-Z-mcDM1 were chosen as the candidates for evaluation in experimental therapy. To connect mcDM1, a C-terminal cysteine was added to Z-ABD and ABD-Z-Z. For Z-ABD, this was achieved by PCR amplification of the gene encoding Z-ABD inserted in the pET21a(+) vector, with primers adding a codon encoding a cysteine to the C-terminus of Z-ABD, alongside NdeI (up-stream) and BamHI (down-stream) restriction enzyme recognition sites. The PCR product was subsequently sub-cloned into the pET26b(+) plasmid using the restriction enzymes NdeI and BamHI. A C-terminal cysteine was added to the gene encoding ABD-Z-Z in pET21a(+) by a modified whole-plasmid mutagenic PCR protocol [34]. Here, a pair of non-overlapping primers, hybridizing at the 3’-end of the ABD-Z-Z-gene, were designed to be used for PCR amplification of the whole plasmid. One of the primers included a codon encoding cysteine placed right after the end of the second gene encoding Z. The linear PCR-product, constituting the whole plasmid, was directly transformed to TOP10 *Escherichia coli (E. coli)* cells (New England Biolabs, Ipswitch, MA, USA). The integrity of the expression cassettes of all plasmids were validated by DNA sequencing by Microsynth (Balgach, Switzerland) or Eurofins (Ebersberg, Germany).

### 2.2. Expression, Purification, and Primary Characterization of the Affibody Constructs

Expression was carried out in *E. coli* BL21*(DE3) (New England Biolabs, Ipswitch, MA, USA). Overnight cultures of plasmid transformed cells were prepared. Next, 10 mL of the overnight cultures were inoculated into pre-warmed 1 L Tryptic Soy Broth (30 g/L) with Yeast Extract (5 g/L) medium at 37 °C. When the OD_600_ was between 0.7 and 0.9, isopropyl β-D-1-thiogalactopyranoside (IPTG; Appolo Scientific, Stockport, UK) was added to a final concentration of 1 mM to induce protein expression. After 4 h of incubation at 37 °C, the cells were harvested by centrifugation.

For purification of Z and Z-Z, the cell pellets were subsequently resuspended in immobilized metal affinity chromatography (IMAC) equilibration buffer (300 mM sodium chloride, 50 mM of sodium phosphate, pH 7.4) with 0.01 mg/mL of deoxyribonuclease I bovine pancreas (Pharmacia Biotech, Uppsala, Sweden) and EDTA-free Halt protease inhibitor cocktail. Cell lysis was performed by a French Press, after which the cell lysates were heated to 60 °C for 10 min to precipitate *E. coli* endogenous proteins. After centrifugation at 16,000 rpm for 20 min at 4 °C, the supernatants were collected and then filtered through a 0.22 μm membrane (Pall Laboratory, Port Washington, NY, USA). Purification was performed by Ni-IMAC. The washing buffer contained 300 mM of sodium chloride, 50 mM of sodium phosphate, and 10 mM of imidazole, pH 7.4. The elution buffer contained 300 mM of sodium chloride, 50 mM of sodium phosphate, and 200 mM of imidazole, pH 7.4. The purifications were carried out on an Äkta Pure instrument (GE Healthcare Life Sciences, Uppsala, Sweden) followed by reverse-phase chromatography (gradient of 20–60% acetonitrile in water with 0.1% trifluoroacetic acid) on a high-performance liquid chromatography instrument using a Zorbax C18 SB column and instrument from Agilent (Santa Clara, CA, USA). Relevant fractions were pooled and lyophilized, and the final products were reconstituted in PBS. The cell pellets of the constructs containing an ABD were resuspended in lysis buffer (50 mM Tris, 0.2 M sodium chloride, 20 mM EDTA, and 0.05% Tween 20, pH 8.0, with 0.01 mg/mL deoxyribonuclease I bovine pancreas and EDTA-free Halt protease inhibitor cocktail). The procedures for cell lysis, centrifugation, and filtration were the same as above. Purification was performed by affinity chromatography on an in-house-made sepharose column with immobilized HSA as described earlier for ABD-containing constructs [35]. Affinity chromatography was followed by reverse-phase chromatography in HPLC mode, lyophilization, and reconstitution as described above.

The purity and molecular weight of the proteins were analyzed by SDS-PAGE using equipment and reagents from Thermo Fisher Scientific. Samples (10 μg) of the reconstituted proteins were mixed with SDS-loading buffer and boiled for 10 min. The samples were subsequently separated on a NuPAGE Bis-Tris protein gel. The marker used was Pageruler pre-stained protein ladder.

### 2.3. Affinity Evaluation of the Affibody Constructs

Surface plasmon resonance (SPR) was used to evaluate the affinity between the constructs and HER2, HSA, MSA, and BSA using a Biacore T200 instrument (Cytiva, Uppsala, Sweden). The extracellular domain of HER2 (Sino biological, Beijing, China) was immobilized on a CM5 chip (Cytiva, Uppsala, Sweden) by amine coupling through *N*-(3-dimethylaminopropyl)-*N*’-ethylcarbodiimide hydrochloride (EDC)/*N*-hydroxysuccinimide (NHS) coupling (Amine coupling kit from Cytiva, Uppsala, Sweden). Similarly, three kinds of serum albumins, namely HSA (Novozymes, Bagsvaerd, Denmark), MSA (Sigma-Aldrich, St. Louis, MO, USA), and BSA (Acros organics, Geel, Belgium), were immobilized on a second CM5 chip. The affinity was measured by single-cycle kinetics, with a flow rate of 50 μL/min, at room temperature. PBST (PBS with 0.005% Tween 20) was used as the running buffer and 10 mM of glycine-HCl, pH 2.0, was used as the regeneration buffer.

### 2.4. Analysis of Cell Proliferation

The SKOV-3 cell line, which overexpresses HER2, was cultured in McCoy’s 5A medium with 10% fetal bovine serum (FBS). The A549 cell line with a low HER2 expression level was cultured in DMEM-high glucose medium with 10% FBS and used as the control. Both cell lines were seeded into 96-well cell culture plates at 500 cells in 100 μL in each well. After incubation for 4 h to allow for cell attachment, the medium was changed into the same medium with 20 nM of the different constructs: Z, Z-ABD, ABD-Z, Z-Z, Z-Z-ABD, Z-ABD-Z, ABD-Z-Z or trastuzumab. The medium was refreshed every three days. On days 10, 20, 27, and 34, the cells were counted using the Cell Counting Kit 8 (Sigma Aldrich, St. Louis, MO, USA). After the cell count, the cells were detached and a fraction was reseeded in new plates.

### 2.5. Labeling of Affibody Constructs with Technetium-99m for In Vitro and In Vivo Characterization

Labeling of the affibody constructs with [^99m^Tc]Tc(CO)_3_ was done similarly to an earlier published procedure [25]. Briefly, tricarbonyl technetium was obtained by incubating freshly eluted [^99m^Tc]TcO_4_ with a CRS kit (PSI, Villigen, Switzerland) for 20 min at 100 °C. Next, 20 μL of the [^99m^Tc]Tc(CO)_3_ solution was incubated with 50 µg of an affibody construct in PBS for 60 min at 60 °C. Labeled constructs were purified on a NAP-5 column (GE Healthcare, Uppsala, Sweden) pre-equilibrated and eluted with 2% BSA in PBS. Silica-impregnated ITLC strips (150–771 DARK GREEN Tec-Control Chromatography strips, Biodex Medical Systems, Shirley, NY, USA), eluted with PBS, were used for the determination of the radiochemical yield and purity of the conjugates. The activity distribution on the ITLC strips was evaluated using a Cyclone Storage Phosphor System (PerkinElmer, Waltham, MA, USA).

### 2.6. In Vitro Characterization of Radiolabeled Affibody Constructs 

The in vitro binding specificity of the radiolabeled constructs was tested on SKOV-3 cells according to Altai et al. [25]. The concentration of the radiolabeled conjugates was 2 nM, while the concentration of the non-radiolabeled conjugates was 200 nM (for receptor pre-saturation), with incubation at 37 °C for 60 min.

Cellular processing of the radiolabeled conjugates was studied using HER2-positive SKOV-3 cells following the method described by Altai et al. [25]. The cells were incubated at 37 °C with radiolabeled affibody conjugates (2 nM). The membrane-bound activity was collected after 5 min of incubation on ice with a 0.2 M glycine buffer containing 4 M of urea, pH 2.5. The internalized activity was collected by further incubation with 1 M of NaOH. The activity in the cell samples was measured using an automated gamma-spectrometer (1480 Wizard; Wallac Oy, Finland). All experiments were performed in triplicates.

### 2.7. Biodistribution of Affibody Constructs Labeled with Technetium-99m in SKOV-3 Xenografted Mice

The biodistribution and tumor targeting of the constructs labeled with [^99m^Tc]Tc(CO)_3_ were studied at 4 and 24 h pi. Mice bearing HER2-positive SKOV-3 xenografts (3 weeks after subcutaneous (sc) inoculation of 10^7^ cells) were used for this study (*n* = 4/group). The xenograft’s size was 0.4 ± 0.2 g. The mice were intravenously (iv) injected with an equimolar amount of radiolabelled conjugates (50 kBq/injection for measurements at 4 h pi and 500 kBq/injection for measurements at 24 h pi) in 100 µL of PBS. The animals were sacrificed by heart puncture after intraperitoneal injection (ip) of an anesthetic solution. The dissected organs and tissue samples were measured for activity content using an automated gamma spectrometer as described above (Section 2.6).

### 2.8. Production of Drug Conjugates

Z-ABD and ABD-Z-Z with C-terminal cysteines were produced as above (Section 2.2) and the lyophilized proteins were reconstituted in PBS (pH 6.6) to a concentration of 5 mg/mL. Next, 5 mM TCEP was used to reduce any oxidized cysteines in the proteins for 1 h at 37 °C. The cytotoxic drug, mcDM1 (Levena Biopharma, San Diego, CA, USA) was dissolved in DMSO to a concentration of 20 mg/mL. The proteins were mixed with mcDM1 to a molar ratio of 3:1 (drug:protein) and incubated for 1 h at room temperature. The drug conjugates were subsequently isolated by RP-HPLC purification as described in Section 2.2. The relevant fractions were lyophilized, followed by reconstitution in PBS.

### 2.9. Evaluation of the Cytotoxic Potential of the Drug Conjugates

Two HER2 over-expressing cell lines, namely SKOV-3 and SKBR3 (cultured in McCoy’s 5A medium with 10% FBS), and two HER2 low/medium expressing cell lines, namely A549 and MCF7 (cultured in DMEM medium with 10% FBS), were utilized to investigate the cytotoxic effects of Z-ABD-mcDM1 and ABD-Z-Z-mcDM1. In total, 5000 cells (2000 cells for SKOV-3) were seeded in the wells of 96-well cell culture plates. Series of concentrations of the drug conjugates were added into the wells and incubated for 72 h at 37 °C in 5% CO_2_. The cell viability was measured using Cell Counting Kit 8.

### 2.10. In Vivo Therapy Using Z-ABD-mcDM1 and ABD-Z-Z-mcDM1 Conjugates in SKOV-3 Xenografted Mice

The therapy was started one week after sc inoculation of 10^7^ cells/mouse. On the day of the first injection, the mice were randomly divided into five groups (*n* = 9–10). Two groups were weekly injected with equimolar amounts (0.36 μmol/kg) of either Z-ABD-mcDM1 (100 µg/injection, 7 nmoles/injection) or ABD-Z-Z-mcDM1 (150 µg/injection, 7 nmoles/injection) in 100 µL of PBS. Control groups were similarly injected with PBS, Z-ABD (93 µg/injection, 7 nmoles/injection), or ABD-Z-Z (143 µg/injection, 7 nmoles/injection). The sizes of the tumors were measured and animal conditions were assessed twice per week according to the guidelines for pain and distress in laboratory animals from the National Institutes of Health (NIH, Bethesda, MD, USA) adopted by Uppsala University. The assessment parameters included the exterior, general conditions, behavior, stress, pain, ataxia, appetite, sores and blistering, eye inflammation, porphyria, the function of urinary and gastrointestinal systems, respiration, and body weight. The tumor volumes were calculated by the formula: V_t_ = ½ (length × width^2^). In total, each mouse received ten injections (if they were not excluded due to large tumor size (>1000 mm^3^), tumor ulceration, or weight loss (15% overall or 10% within one week). The endpoint of the study was 90 days after the first injection. After the mice were sacrificed, the tumors, livers, and kidneys were investigated for pathological changes.

## 3. Results

### 3.1. Expression and Purification of the Affibody Constructs

**Figure 1:**
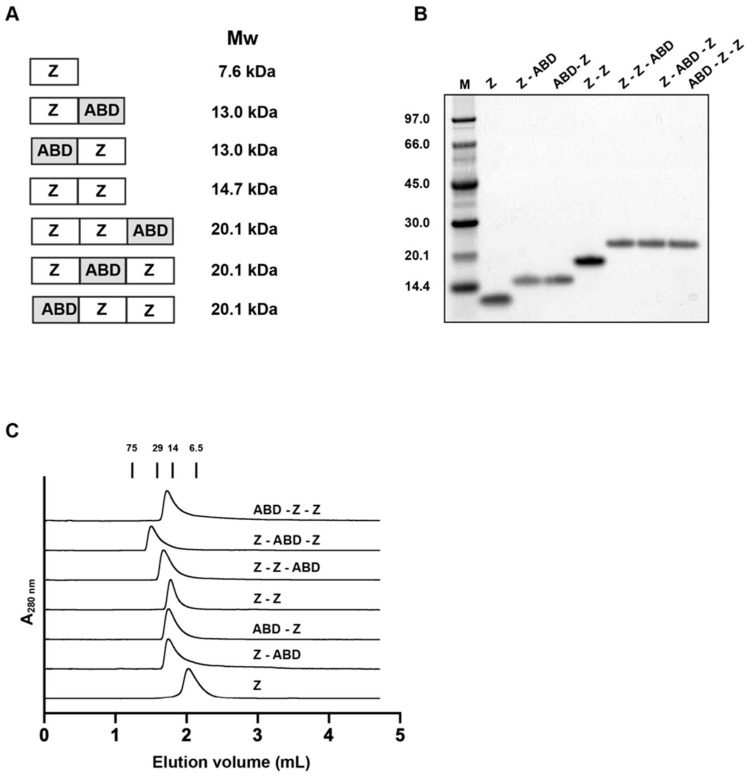
Schematic representation and primary characterization of the seven constructs. (**A**) The composition and theoretical molecular weight of the seven constructs are shown. Z and ABD represent Z_HER2:2891_ and ABD_035_, respectively. A linker with the amino acid sequence Gly_4_Ser was included between each Z and ABD domain. (**B**) The two-step purified proteins were analyzed by sodium dodecyl sulphate–polyacrylamide gel electrophoresis (SDS-PAGE) under reducing conditions. The numbers to the left of the gel correspond to the molecular weight (in kDa) of the marker proteins loaded in lane M. (**C**) The chromatograms from the size exclusion chromatography analyses are shown. The numbers above the chromatograms correspond to the elution volume of marker proteins with the indicated molecular weights (in kDa).

The composition of the seven affibody carriers are shown in Figure 1A. All proteins were produced in *Escherichia coli (E. coli)* and then purified by liquid chromatography. The purities of the constructs were validated by SDS-PAGE (Figure 1B). They were all of essentially the right size and of high purity. Moreover, the proteins’ molecular weights were measured by liquid chromatography-electrospray ionization mass spectrometry and are displayed in Table 1. The difference between the theoretical and measured molecular weight was found to be below 1 Da. Finally, analytical size exclusion chromatography under native conditions was performed to investigate if the proteins were forming higher-order structures or if there were any degradation products (Figure 1C). Since a single peak was found in each chromatogram for each construct, with essentially the expected molecular weight, it was deduced that the constructs were present as monomers when in PBS.

### 3.2. Evaluation of Affinities

The affinities of the affibody constructs to HER2, HSA, and MSA were investigated by surface plasmon resonance (SPR) analysis. The equilibrium dissociation constants (K_D_-values) were calculated assuming a 1:1 Langmuir model and fitting accuracies were overall very high (Table 2 and Supplementary Figure S1). The affinity of Z and Z-ABD towards HER2 was similar, with K_D_ values of the interactions of 0.5 and 0.4 nM, respectively. By contrast, the third construct with a single affibody-domain, ABD-Z, had a five-fold weaker affinity towards HER2. The apparent affinity of Z-Z towards HER2 was the strongest with a K_D_ of 0.03 nM. The three constructs including two Z_H_ domains and an ABD at different positions, had apparent affinities between 0.7 and 1 nM. These results show that the seven constructs all have a high affinity to HER2. Furthermore, the affinities to three types of SA from different species were determined. As expected, the fusion proteins lacking an ABD, namely Z and Z-Z, did not interact with any of the SAs (Table 2). Z-ABD had the highest affinity to HSA and MSA among the constructs, with K_D_-values of 0.1 and 3.1 nM, respectively. The weakest affinity towards HSA and MSA was found for ABD-Z, with K_D_-values of 1.0 and 6.2 nM, respectively. The fusion proteins consisting of ABD and two Z-domains had similar affinities to the SAs, with K_D_-values of around 0.6 nM for HSA and 3 to 4 nM for MSA. All ABD-fused constructs could thus bind to both HSA and MSA, in all cases with a stronger affinity towards HSA. The interaction of the fusion proteins with the surface, with immobilized BSA, did not give any measurable signal, which was expected since the interaction has previously been shown to be weak (K_D_ > 1 μM) [30].

### 3.3. Effect on Cell Proliferation

**Figure 2:**
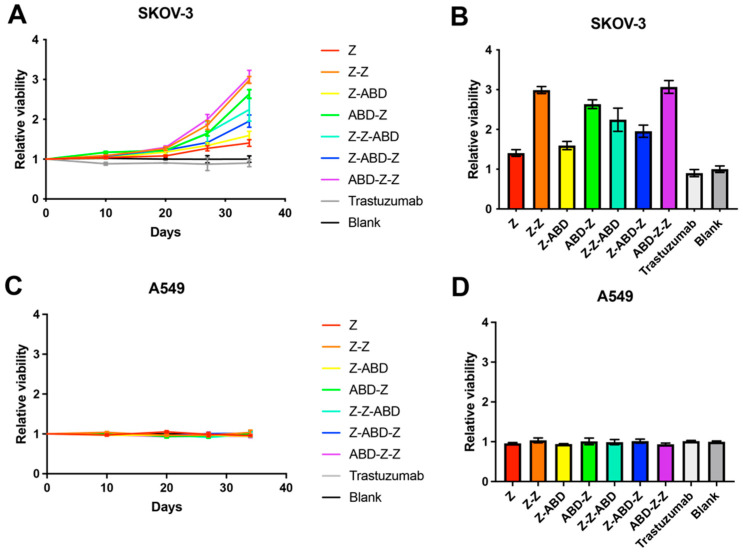
Effects on cell proliferation by the affibody constructs. (**A**,**C**) The relative viability of SKOV-3 cells (**A**) and A549 cells (**C**) was measured as a function of time. The relative viability was calculated by dividing each culture’s viability with that of the blank, where cells were grown in complete medium without the addition of any construct. (**B**,**D**) The columns represent the relative viability measured at day 34 for SKOV-3 cells (**B**) and A549 cells (**D**). All the data points are based on triplicate measurements and the error bars correspond to 1 SD.

The effect on cell proliferation was evaluated in a high-HER2-expressing cell line (SKOV-3) and a medium/low HER2-expressing cell line (A549) and the results are shown in Figure 2. The cells were cultured in complete medium supplemented with 20 nM of one of the constructs. For the SKOV-3 cell line, no significant difference in cell number was found for any of the constructs for the first 20 days of cultivation (Figure 2A). However, after day 20, the cell numbers started to diverge. All variants with two Z domains promoted cell proliferation to different extents compared to the control, where no construct was added. The constructs with one Z domain promoted SKOV-3 proliferation to different extents. Z and Z-ABD had a minor effect, while ABD-Z promoted proliferation more strongly (Figure 2B). Trastuzumab was included as a control and did not affect cell proliferation significantly. The proliferation of A549 cells, with medium/low expression of HER2, was not affected by any of the seven constructs or trastuzumab (Figure 2C,D).

### 3.4. Characterization of Affibody Constructs Labeled with Technetium-99m

All conjugates were successfully labeled with [^99m^Tc]Tc(CO)_3_ and after size-exclusion chromatography purification, demonstrated a radiochemical purity >93%, determined by ITLC. All constructs preserved a specific binding to HER2, as demonstrated by blockable-binding to HER2-expressing cells for all conjugates (Supplementary Figure S2). Furthermore, the association and cellular processing of the conjugates were determined as a function of time. All conjugates demonstrated rapid binding to HER2-positive cells (Supplementary Figure S3). The cell association activity reached a maximum within 2–4 h of continuous incubation for all conjugates, except for [^99m^Tc]Tc-Z-ABD and [^99m^Tc]Tc-Z-Z-ABD, for which the cell-associated activity continued to grow. With time, all constructs demonstrated a slowly increased internalized fraction of activity, which was somewhat higher for the dimeric constructs. At 24 h, the internalized fraction for monomers was 25–30%, and for dimers, it was 30–50% of the total cell-associated activity. 

### 3.5. Biodistribution of the Affibody Constructs in Tumor-Bearing Mice

**Figure 3:**
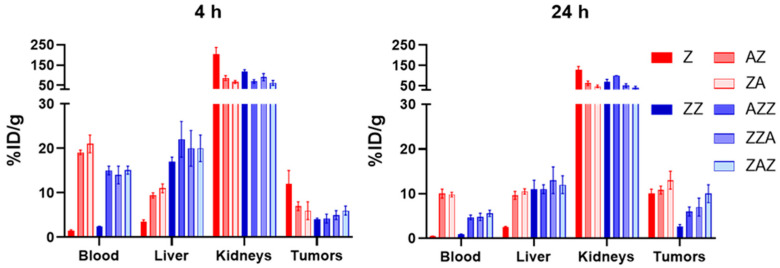
Biodistribution of HER2-targeting conjugates labeled with technetium-99m in mice bearing HER2-positive SKOV-3 xenografts. Data are presented as an average value from the selected organs and tumor ± 1 SD, *n* = 4.

The biodistribution of the affibody constructs was studied in SKOV-3 xenografted mice (Figure 3 and Supplementary Table S1). Both affibody constructs lacking the ABD, namely ^99m^Tc-Z and ^99m^Tc-Z-Z, demonstrated rapid blood clearance via renal elimination with a high degree of reabsorption in the kidneys. The activity concentration in blood was below 2.5%ID/g at 4 h pi for these conjugates. The dimeric affibody construct, specifically [^99m^Tc]Tc-Z-Z, had a five-folds higher uptake in the liver compared to the monomeric [^99m^Tc]Tc-Z. When coupled to the ABD, both monomeric and dimeric constructs had longer retention in blood than [^99m^Tc]Tc-Z and [^99m^Tc]Tc-Z-Z. The blood retention was somewhat longer for the conjugates including one Z domain than for the conjugates including two Z domains. At 4 h pi, [^99m^Tc]Tc-Z-ABD and [^99m^Tc]Tc-ABD-Z had an activity concentration in the blood of ~20%ID/g, and at 24 h pi, the activity was ~10%ID/g. The activity concentration for [^99m^Tc]Tc-Z-Z-ABD, [^99m^Tc]Tc-Z-ABD-Z, and [^99m^Tc]Tc-ABD-Z-Z was lower, around 15 and 5%ID/g at 4 and 24 h pi, respectively. All ABD-coupled conjugates demonstrated a high activity uptake in excretory organs, namely in the liver and kidneys. The hepatic uptake was two-folds higher for [^99m^Tc]Tc-Z-Z-ABD, [^99m^Tc]Tc-Z-ABD-Z, and [^99m^Tc]Tc-ABD-Z-Z at 4 h pi. The ABD-fused constructs demonstrated a comparable level of renal uptake, which was significantly lower than for the non-ABD-fused fusion proteins [^99m^Tc]Tc-Z and [^99m^Tc]Tc-Z-Z. All constructs were taken up by the HER2-expressing SKOV-3 tumors already at 4 h pi. Among the tested constructs, at 4 h pi, [^99m^Tc]Tc-Z demonstrated the highest activity uptake in tumors and the activity was not washed-out up to 24 h pi. Furthermore, [^99m^Tc]Tc-Z-Z had a three-folds lower tumor uptake than [^99m^Tc]Tc-Z at 4 h pi. At 24 h pi, the activity uptake of [^99m^Tc]Tc-Z-Z was 30% lower than that at 4 h pi. Conversely, the activity uptake in tumors increased by 50–100% between 4 and 24 h pi for the ABD-fused conjugates. The highest activity uptake among the ABD-fused conjugates at 24 h pi was observed for [^99m^Tc]Tc-Z-ABD. Generally, ABD-fused constructs with two affibody domains had a lower activity uptake in tumors than the ABD-fused constructs with only one affibody domain at both time points. Overall, among the ABD-fused constructs, the constructs with only one Z domain had more favorable biodistribution patterns than the constructs with two Z domains, with significantly higher uptake in tumors, better blood retention, and lower hepatic uptake.

### 3.6. Production and Characterization of Affibody-DM1 Conjugates

**Figure 4:**
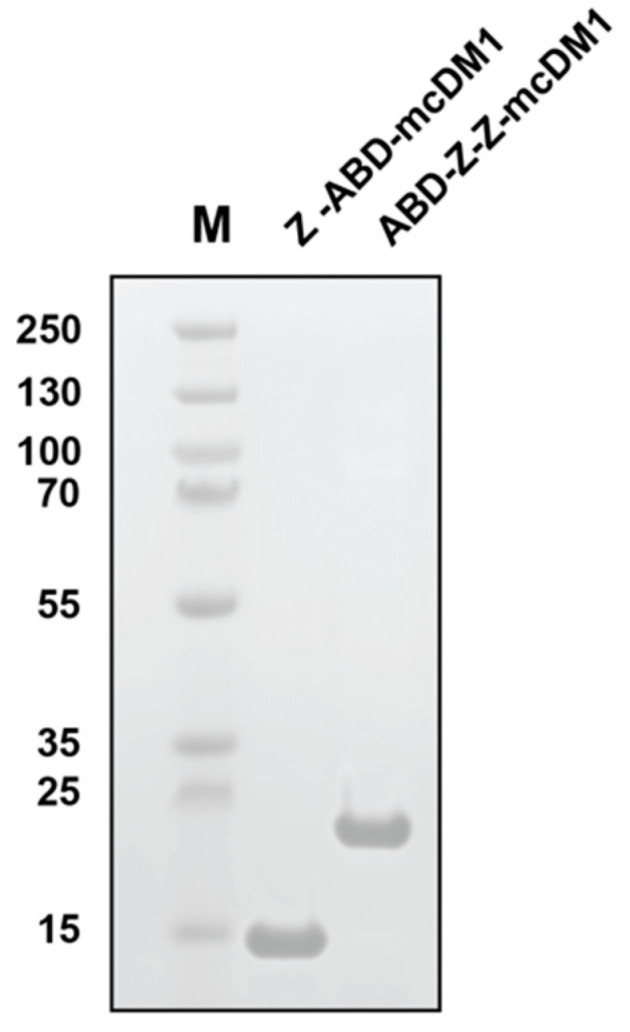
SDS-PAGE analysis of the two-step purified drug conjugates Z-ABD-mcDM1 and ABD-Z-Z-mcDM1 under reducing conditions. The numbers to the left of the gel correspond to the molecular weight (in kDa) of the marker proteins loaded in lane M.

To evaluate the effect of using a DM1-carrier for experimental therapy that promotes cell proliferation more strongly with a carrier that promotes cell-proliferation more weakly, Z-ABD (weak) and ABD-Z-Z (strong) were chosen. The genes encoding both carriers were re-cloned, adding a codon coding for a cysteine amino acid in the C-terminus. The genes were expressed in *E. coli.*, purified by column chromatography, and mcDM1 was site-specifically conjugated to the C-terminal cysteine, yielding Z-ABD-mcDM1 and ABD-Z-Z-mcDM1. After the final purification step, samples of the two drug conjugates were loaded on an SDS-PAGE gel, which showed pure conjugates with essentially the expected molecular weight (Figure 4). Mass spectrometry analysis showed values of the molecular weight that differed by less than 1 Da from the theoretical values (Table 3). Affinity determination by a real-time biosensor showed that the affibody-mcDM1 conjugates had a similar affinity to HER2, HSA, and MSA (Table 4 and Supplementary Figure S4), comped to the corresponding fusion proteins without DM1 (Table 2). Thus, DM1 modification did not influence the affinities to HER2, HSA, or MSA significantly. 

**Figure 5:**
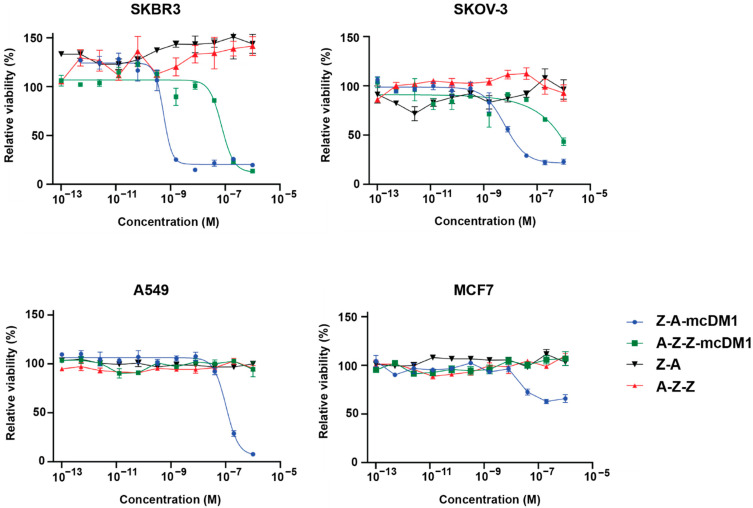
Determination of the cytotoxic potential. Dilution series of the affibody-DM1 conjugates and the corresponding non-toxic controls were incubated with different tumor cell lines. The curves show the relation between the relative viability of cells and the concentration of the constructs. Cells incubated in complete medium without any construct were set to 100%. All data points are based on triplicate measurements and the error bars correspond to SD.

The cytotoxic potential of the conjugates was measured by culturing several cell lines in complete medium supplemented with series of concentrations of the drug conjugates or non-toxic controls, namely Z-ABD and ABD-Z-Z, followed by measurement of cell viability (Figure 5). The IC50-value for Z-ABD-mcDM1 was 100-folds lower than the IC50-value for ABD-Z-Z-mcDM1 on SKBR3 and SKOV-3 cells, with high HER2 expression (Table 5). Z-ABD-mcDM1 showed a weaker IC50-value towards A549 cells with low/medium HER2 expression compared to the high-HER2-expressing cell lines. ABD-Z-Z-mcDM1 did not affect the viability of the A549 cell line. Furthermore, Z-ABD-mcDM1 and ABD-Z-Z-mcDM1 did not affect the viability of the MCF7 cell line with low HER2 expression. The non-toxic control constructs, namely Z-ABD and ABD-Z-Z, did not significantly affect the viability of any of the cell lines.

### 3.7. In Vivo Therapy Using Affibody-DM1 Conjugates in SKOV-3 Xenografted Mice

**Figure 6:**
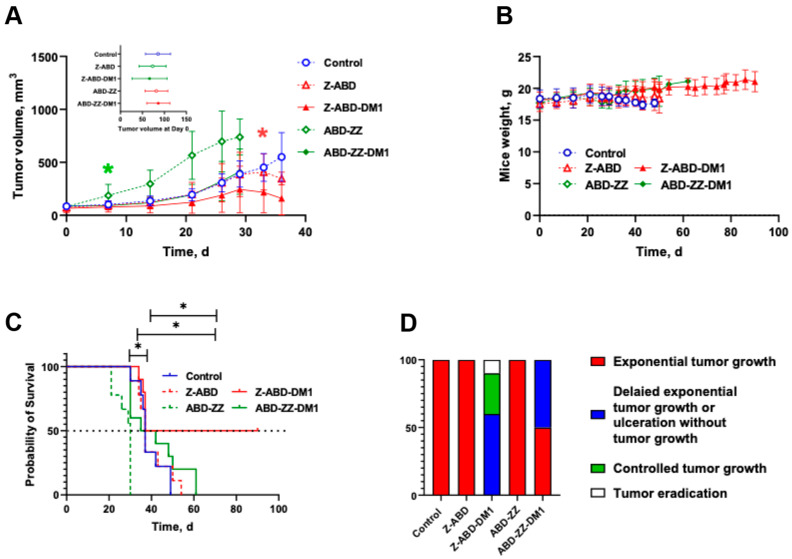
In vivo experimental therapy using Z-ABD-mcDM1, ABD-Z-Z-mcDM1, and the controls Z-ABD, ABD-Z-Z_,_ and BSA in PBS. Treatment was started at day 0 and continued weekly for 10 weeks or to the humane end point (xenograft volume over 1 cm^3^ or tumor ulcerated). (**A**) The average tumor volume of each group as a function of time ± SD. Curves were stopped when 30–33% of the mice in a group were euthanized. (**B**) The average mice weight in groups as a function of time ± SD. (**C**) The survival until euthanasia criteria of the xenografted mice. (**D**) The therapy output. * corresponds to significant difference (*p* < 0.05).

Next, the potency of the drug conjugates for therapy was investigated in a xenograft murine model. The experiment was performed in mice bearing HER2-positive SKOV-3 xenografts and the growth of the tumors are presented in both Figure 6A and Supplementary Figure S5. At the start of the experiment, the tumor volumes were 77 ± 32 mm^3^ and there was no significant difference between the groups (*n* = 9–10, *p* = 0.5597, R^2^ = 0.06717). The mice were treated with weekly injections. The treatment was well-tolerated and no visible side effects were observed. The animal’s weight showed a tendency to increase until the tumor size approached 1 cm^3^ (Figure 6B). 

The tumors in the control group, receiving injections of 0.5% BSA in PBS, demonstrated an exponential growth typical for SKOV-3 xenografts and the median survival time in this group was 37 days (Figure 6C). On day 49, all mice in this group were sacrificed. 

The tumor volumes in the group treated with the non-toxic control construct ABD-Z-Z were significantly larger than in all other groups already one week after the treatment’s start (*p* < 0.0001). The median survival was 30 days for this group, which was significantly shorter than for all other groups including the control group (*p* = 0.0002). Eight out of nine mice were sacrificed by day 30 due to a large tumor size and/or severe ulceration. Conversely, the mcDM1-derivative of ABD-Z-Z, namely ABD-Z-Z-mcDM1, significantly slowed down the tumor growth and improved median survival in comparison with its non-toxic control construct ABD-Z-Z. The median survival was 37 days for this group, which was the same as for the control group receiving injections of BSA in PBS.

The group treated with the non-toxic control, Z-ABD, had a tumor growth and median survival time similar to the control group receiving BSA in PBS (38.5 days, *p* = 0.5703). By contrast, the derivatization with mcDM1, yielding Z-ABD-mcDM1, appreciably slowed down tumor growth. In one mouse, the tumor that initially had a volume of 156 mm^3^ was eradicated on day 26. On day 33, the tumors in this group were significantly smaller in comparison with the control groups receiving BSA in PBS (*p* = 0.009) or Z-ABD (*p* = 0.04). A further decrease in the tumor volumes was detected on day 36 and was accompanied by ulceration in some tumors, which was an ultimate exclusion criterion in this study (Supplementary Figure S5). After day 36, tumor growth in the remaining four mice was under control until the end of the experiment on day 90. Tumor re-growth was observed in one mouse in this group after treatment was stopped. The median survival time in the group treated with Z-ABD-mcDM1 was 63.5 days, which was significantly longer than in the other groups receiving BSA in PBS (*p* = 0.05), Z-ABD (*p* = 0.04), or ABD-Z-Z-mcDM1 (*p* = 0.03; Figure 6C). Therapy output (animals that exhibited tumor growth delay, controlled tumor growth, or eradication of the established tumor) was significantly better for the group treated with Z-ABD-mcDM1 than for the other groups (*p* < 0.0001, Χ^2^(2) = 90.91 compared to the group treated with ABD-Z-Z-mcDM1 and *p* < 0.0001, Χ^2^(2) = 200 compared to the groups treated with Z-ABD, ABD-Z-Z, or the control group; Figure 6D).

Tumors, livers, and kidneys were dissected and examined for pathological changes (Supplementary Figure S6). Histological examination of the liver and kidneys showed no significant histological damage: in all groups, parenchymal cells in both organs remained normal and there were no signs of toxic damage. In the control group, weak variations in hepatocyte nuclei were noted. In the treated groups, hepatocytes in some cases had different nuclear sizes (mainly in groups treated with Z-ABD-mcDM1). In the same groups, signs of fatty change hepatocytes of a weak degree were noted. Tumors in all groups were located in the dermis, widely varied in size, and had an expansive type of growth. Mitotic activity was moderate to high with atypical mitotic figures. Several tumors in groups treated with Z-ABD-mcDM1, ABD-Z-Z, and ABD-Z-Z-mcDM1 showed mild fibrosis. The death of tumor cells occurred in the form of apoptosis or necrosis of single to few cells, but also in the form of small necrotic areas. Large necrosis was found in the central zone of one examined tumor in the group treated with ABD-Z-Z. In some tumors in groups treated with the affibody conjugates or the controls without DM1, inflammatory cell infiltrates were found. Inflammatory cellular reactions varied in intensity and were predominantly revealed in the peripheral zones of the tumors. No metastatic foci were found in the examined healthy tissues (liver and kidneys).

## 4. Discussion

Mitosis is a vulnerable stage of the cell cycle and if the mitotic spindle does not form properly, viable progeny will not form. Cancer cells have a higher rate of proliferation than most normal cells in the body and they are therefore more susceptible to agents disrupting microtubule formation than normal cells. Agents interfering with proper microtubule formation have been used for cancer therapy for a long time, such as, for example, taxanes and vinca alkaloids [36]. More recently, the delivery of microtubule disrupting agents coupled to tumor-targeting antibodies has been shown efficacious, as demonstrated, for example, by trastuzumab emtansine for the treatment of breast cancer [36]. However, might the effectiveness of microtubule-disrupting agents be influenced by the proliferation rate of cancer cells, where cells with a higher rate of proliferation are more sensitive?

In this study, we have addressed this question by investigating the effectiveness of HER2-targeted, microtubule-disrupting drug conjugates that induce cancer cell proliferation to different extents. Seven HER2-binding affibody fusion proteins, composed of one or two affibody domains (Z) with or without an ABD-domain, were constructed (Z, Z-ABD, ABD-Z, Z-Z, Z-Z-ABD, Z-ABD-Z, and ABD-Z-Z). These proteins could be produced in *Escherichia coli* and were recovered with a high yield after a two-step purification procedure. All constructs had a similar apparent affinity to HER2 in vitro in the low to the sub-nanomolar range (Table 4). The weakest affinity was found for ABD-Z and a possible explanation is that a free N-terminus of Z_HER2_ is necessary for proper interaction with HER2. However, for the constructs including two Z_HER2_ domains, the same loss in affinity was not observed and the affinities to HER2 for ABD-Z-Z, Z-Z-ABD, and Z-ABD-Z were similar. According to the measured interactions with SAs, all constructs that included an ABD domain had similar affinities for HSA in the sub-nanomolar range and for MSA in the low nanomolar range. Collectively, these results show that the binding properties of ABD to SA were not affected by N or C-terminal extension with an affibody molecule.

Furthermore, the influence of the constructs on cell proliferation was investigated. All seven constructs, both monomeric and dimeric, promoted proliferation of the SKOV-3 cells and Z-Z, ABD-Z-Z_,_ and ABD-Z had the strongest effect (Figure 2). In vitro, the effect was only visible after day 20 and it was therefore not surprising that no effect was seen for Z-ABD or ABD-Z-Z when they were used as non-toxic controls during the determination of the cytotoxic potential of the drug conjugates (Figure 5) since the cells were only cultivated for three days in that experiment. A similar growth-stimulating effect was previously observed when incubating SKOV-3 cells with the dimeric HER2-binding affibody construct Z_HER2:4_-Z_HER2:4_ [31]. Interestingly, the effect appears to be cell line-dependent and the reverse effect, namely growth inhibition, has previously been observed when treating SKBR3 cells with the same dimeric construct [18,31]. Earlier, Jost and co-workers investigated the capacity of another class of targeting agents, namely DARPins, to attenuate or promote the growth of HER2-expressing cells [17]. In that case, the linker length played a major role and the same DARPins connected with linkers of different lengths could promote or attenuate proliferation possibly due to the fact that they arranged the receptors in a position relative to each other that was favorable or unfavorable for the triggering of intracellular pathways leading to the promotion of proliferation.

Constructs radiolabeled with technetium-99m were used to determine the biodistribution pattern in mice. All constructs accumulated in the tumors. The construct Z-ABD had the most favorable biodistribution, with high tumor uptake accompanied by low hepatic uptake and long retention in blood, compared to the other constructs. The long retention in blood resulted in the highest increase in tumor activity uptake between 4 and 24 h pi. However, all constructs had a significant uptake in the liver and kidneys. This uptake could potentially be alleviated by adding hydrophilic and negatively charged amino acids, such as three to six glutamic acids, close to where DM1 is attached on the carrier, as we have demonstrated in an earlier study [37]. Additionally, there was no significant difference in the tumor uptake of Z, Z-ABD, and ABD-Z at 24 h, even though the ABD prolongs the plasma half-life; however, the uptake of Z in the kidneys was significantly higher than the other two. This result suggests that the ABD might not be necessary and a future line of research could be to investigate the therapeutic efficacy and side-effects of constructs with and without the ABD.

Two conjugates that demonstrated the most favorable biodistribution profiles were chosen for further studies to investigate how stimulation of proliferation influences the therapeutic efficacy of mcDM1 in HER2-expressing cells. Those chosen were the monomeric Z-ABD, which weakly promoted cell proliferation, and a dimeric variant, ABD-Z-Z, which promoted cell proliferation more strongly. The two fusion proteins were conjugated with mcDM1, resulting in the drug conjugates Z-ABD-mcDM1 and ABD-Z-Z-mcDM1. The production of the drug conjugates was straight-forward and the biochemical characterization of the final products showed homogenous drug conjugates with one DM1 molecule per protein carrier. The addition of mcDM1 did not significantly affect the binding of the drug conjugates towards their intended targets, namely HER2, HSA, and MSA. The in vitro cytotoxicity analysis (Figure 5) showed that Z-ABD-mcDM1 was more potent towards both tested cell lines with high HER2 expression, namely towards SKBR3 and SKOV-3, than ABD-Z-Z-mcDM1. Since the rates of internalization for both protein carriers were similar (Supplementary Figure S3), the results suggest that the architecture, including the molecular size, influences the in vitro potency in the absence of an inhibitory or stimulating effect on proliferation. In particular, it appears that a smaller size results in a more potent compound, something we have also observed in an earlier study [38]. In the earlier study, it was also found that addition of hydrophilic glutamic acids next to the cysteine where DM1 was attached resulted in a lower uptake in, e.g., the liver, probably due to a shielding of the hydrophobicity of DM1. In future studies, it would be interesting to investigate how the addition of glutamic acids near the site of DM1 attachment on A-Z-Z affects the behavior of this drug conjugate. 

To further analyze the ability of Z-ABD-mcDM1 and ABD-Z-Z-mcDM1 to act on HER2-overexpressing cells, they were given as experimental therapy to mice with SKOV-3 xenografts. The results demonstrated that the treatment of HER2-positive tumors with the mcDM1-coupled conjugates significantly decreased tumor growth in comparison with the groups treated with the same protein carrier lacking mcDM1. However, ABD-Z-Z-mcDM1 just marginally improved the mean survival time in comparison with the control group receiving BSA in PBS. This could probably be explained by the fact that the dimeric construct without mcDM1 stimulated tumor growth in the mice. 

In vitro, incubation of HER2-positive cells with Z-ABD did show a slight increase in cell proliferation (Figure 2) but no proliferative effect was observed in vivo (Figure 6). The monomeric drug conjugate Z-ABD-mcDM1 demonstrated a pronounced inhibiting effect on tumor growth and significantly prolonged the average survival time. It is interesting to note that while tumors in 40% of the mice treated with Z-ABD-mcDM1 demonstrated an early response to the treatment, other tumors exhibited growth. These growing tumors drastically decreased in volume after four therapy cycles that, unfortunately, were accompanied by tumor ulceration in a majority of the mice, which then had to be excluded. A single mouse in this sub-group that did not develop an ulcer when the tumor collapsed further demonstrated a stable disease similar to the sub-group of responders (Supplementary Figure S5). 

The post-mortem examination of the excretory organs, namely the livers and kidneys, that demonstrated a high uptake of affibody constructs (Figure 3) did not detect any significant pathological changes related to the toxicity of DM1. The fatty change hepatocytes (of a weak degree) noted in the group treated with Z-ABD-mcDM1 could be attributed to the difference in mice age since this group had longer survival time (Figure 6).

The cumulative molar drug dose in this study was equal to the dose used in our previous study [25], although given during a longer period of time. The therapeutic efficacy of ABD-Z-Z-mcDM1 was weaker than for a similar conjugate tested by Altai et al. (Z-Z-ABD-mcDM1). The median survival time in this study increased by 23% for the group treated with ABD-Z-Z-mcDM1 in comparison with the group treated with ABD-Z-Z, while in our previous study [25], the median survival time for the group treated with Z-Z-ABD-mcDM1 increased by 176% in comparison with Z-Z-ABD. This is in good agreement with the difference in the in vitro stimulating effect for ABD-Z-Z and Z-Z-ABD (Figure 2), where ABD-Z-Z simulates cell growth more than Z-Z-ABD. In the presented study, we have proved the hypothesis that the domain permutation of anti-HER2 affibody-based drug conjugates influences their therapeutic effect. The data presented in this study clearly demonstrate that for HER2-positive cells, additional stimulation of cell proliferation does not improve the therapeutic effect in vivo. In future studies, it would be interesting to investigate how the drug conjugates affect more slowly diving cells, such as cancer stem cells, which are notoriously difficult to eliminate with microtubule-disrupting agents [39].

## 5. Conclusions

In conclusion, in this study, we have designed and studied seven different constructs based on the HER2-targeting affibody molecule Z_HER2:2891_ and albumin-binding domain ABD_035_. We have demonstrated that the assembling of mono and di-valent anti-HER2 constructs significantly affects HER2-induced proliferation. We found that the therapeutic effect of the mertansine-derivate, mcDM1, was significantly decreased by stimulation of the proliferation induced by a dimeric affibody-based delivery vector. We found that Z_HER2_-ABD was the most promising format for efficient DM1 action on HER2-expressing tumors considering its favorable biodistribution pattern and cytotoxic potential. 

## Figures and Tables

**Table 1:** Theoretical and measured values of the proteins’ molecular weights.

Affibody Carrier	Theoretical Mw (Da)	Measured Mw (Da) ^a^	Difference (Da)
Z	7642.6	7641.8	−0.8
Z-ABD	13,049.9	13,048.9	−1.0
ABD-Z	13,049.9	13,048.9	−1.0
Z-Z	14,652.4	14,651.6	−0.8
Z-ABD-Z	20,059.7	20,059.5	−0.2
ABD-Z-Z	20,059.7	20,059.5	−0.2
Z-Z-ABD	20,059.7	20,059.5	−0.2

^a^ Measurement by liquid chromatography mass spectrometry (LC-MS).

**Table 2:** The affinities between the constructs and HER2 or SAs.

Affibody Carrier	HER2 (nM)	HSA (nM)	MSA (nM)
Z	0.45 ± 0.04 ^a^	N.D. ^b^	N.D.
Z-ABD	0.41 ± 0.01	0.14 ± 0.03	3.1 ± 0.2
ABD-Z	2.3 ± 0.1	0.95 ± 0.03	6.2 ± 1.2
Z-Z	0.031 ± 0.007	N.D.	N.D.
Z-ABD-Z	0.71 ± 0.02	0.62 ± 0.27	3.6 ± 0.3
ABD-Z-Z	0.99 ± 0.07	0.62 ± 0.02	3.3 ± 0.2
Z-Z-ABD	0.84 ± 0.02	0.62 ± 0.03	3.6 ± 0.2

^a^ Each value is the average of three independent measurements ± 1 SD. Representative sensorgrams used for the measurements are shown in Supplementary Figure S1. ^b^ Not determined.

**Table 3:** Theoretical and measured values of the drug conjugates’ molecular weights.

Affibody Carrier	Theoretical Mw (Da)	Measured Mw (Da) ^a^	Difference (Da)
Z-ABD-DM1	13,996.4	13,995.2	−1.2
ABD-Z-Z-DM1	21,006.2	21,005.6	−0.6

^a^ Measurement by liquid chromatography mass spectrometry (LC-MS).

**Table 4:** The equilibrium dissociation constants between the constructs and HER2 or SAs.

Affibody Carrier	HER2 (nM) ^a^	HSA (nM)	MSA (nM)
Z-ABD-DM1	0.33 ± 0.07	0.091 ± 0.005	0.279 ± 0.007
ABD-Z-Z-DM1	1.33 ± 0.04	0.847 ± 0.009	0.18 ± 0.03

^a^ Each equilibrium dissociation constant (K_D_ value) was measured in triplicates based on the sensorgrams in Supplementary Figure S4.

**Table 5:** Cytotoxic potential of the drug conjugates.

Cell Line	IC_50_ (nM) ^a^	
	Z-A-mcDM1	A-Z-Z-mcDM1
SKBR3	0.6	74
SKOV-3	6	ND ^b^
A549	100	ND

^a^ Values are calculated from data plotted in Figure 5 and are based on triplicate experiments. ^b^ Not determined.

## Data Availability

All data is contained within the manuscript.

## Supplementary Materials

The following are available online at www.mdpi.com/article/10.3390/pharmaceutics13111974/s1, Figure S1: Measurement of the interactions between the affibody constructs and HER2 (A), HSA (B), and MSA (C) by SPR analysis; Figure S2: In vitro specificity test for affibody conjugates labeled with [99mTc]Tc(CO)_3_ on high-HER2-expressing SKOV-3 cells; Figure S3: Cellular processing of the affibody constructs; Figure S4: Measurement of the affinity of the drug conjugates; Figure S5: Tumor growth curves in individual mice; Figure S6: Results of the pathological examination; Table S1: Biodistribution of the affibody-based fusion proteins labeled with technetium-99m at 4 and 24 h after IV injection in BALB/c nu/nu mice bearing HER2-positive SKOV-3 xenografts.
