# Supplementary material for: The Influence of Domain Permutations of an Albumin-Binding Domain-Fused HER2-Targeting Affibody-Based Drug Conjugate on Tumor Cell Proliferation and Therapy Efficacy
Source: Pharmaceutics. 2021 Nov 21;13(11):1974. doi: 10.3390/pharmaceutics13111974 (PMC8617914; doi:10.3390/pharmaceutics13111974)
Supplement: Supplementary file 1 [file pharmaceutics-13-01974-s001.zip › pharmaceutics-1423402-supplementary.pdf]

pharmaceutics

MDPI

Supplementary Materials: The Influence of Domain-Permutations of an Albumin-Binding Domain-Fused HER2-Targeting Affibody-Based Drug Conjugate on Tumor Cell Proliferation and Therapy Efficacy

Wen Yin, Tianqi Xu, Mohamed Altai, Maryam Oroujeni, Jie Zhang, Anzhelika Vorobyeva, Olga Vorontsova, Sergey V. Vtorushin, Vladimir Tolmachev, Torbjörn Gräslund and Anna Orlova

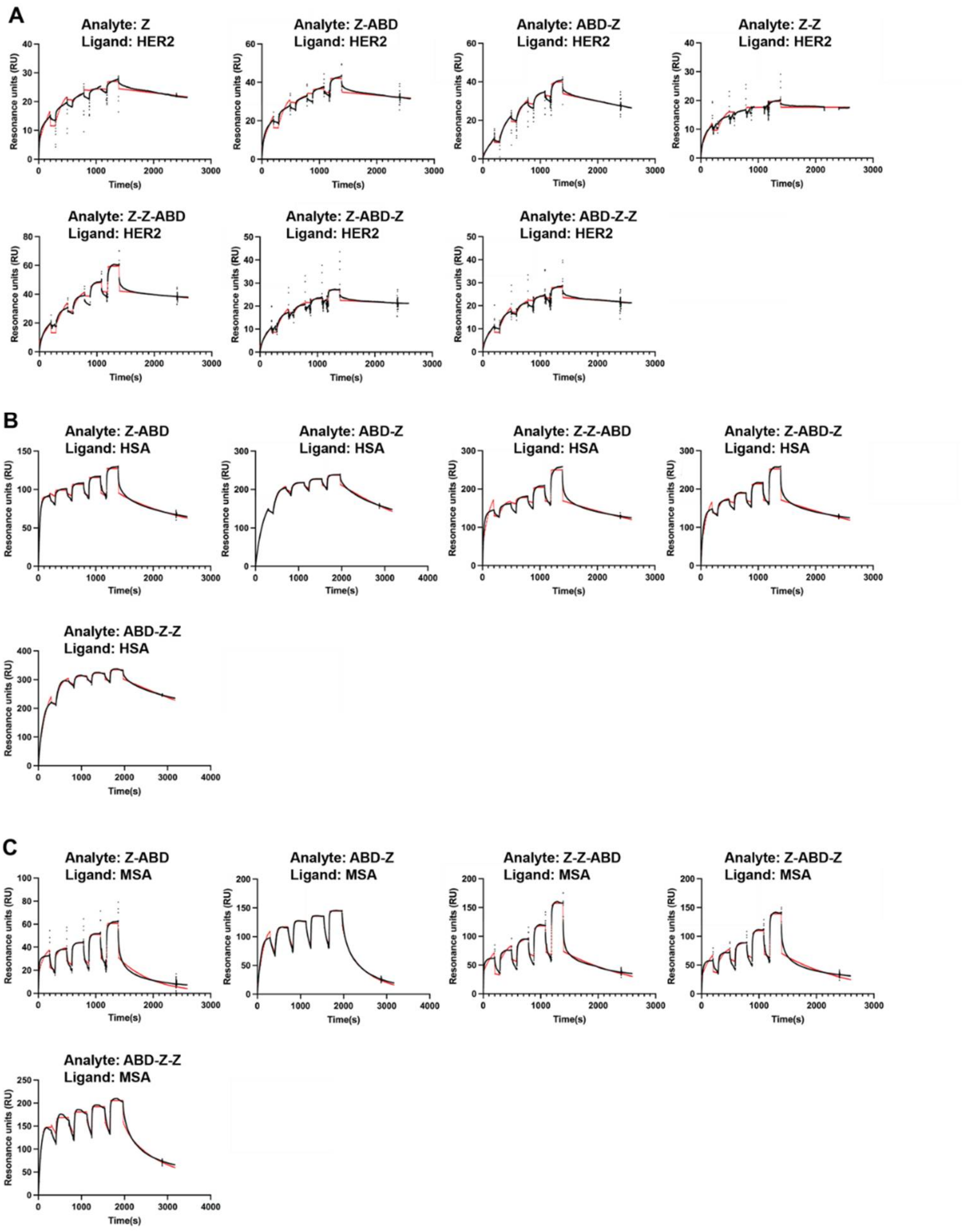

Figure S1. Measurement of the interactions between the affibody constructs and HER2 (**A**), HSA (**B**), and MSA(**C**) by SPR analysis. Dilution series of the constructs were sequentially injected in single-cycle analysis mode. The black curves correspond to representative sensorgrams recorded during the analysis. The red curves correspond to the theoretical curves drawn based on the kinetic parameters of each interation.

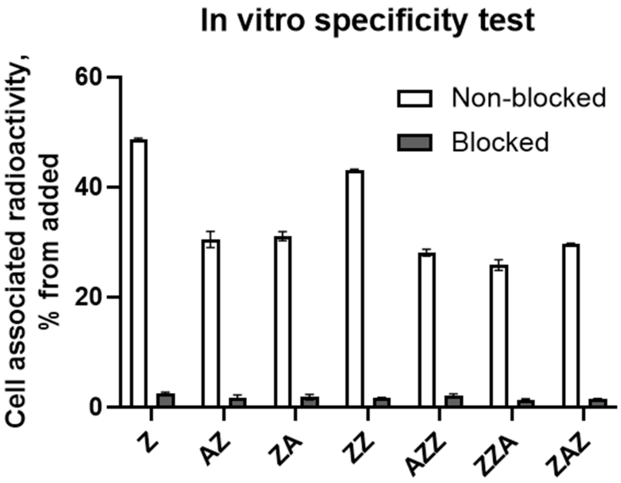

Figure S2. In vitro specificity test for affibody conjugates labeled with [99mTc]Tc(CO)$_3$ on HER2-high expressing SKOV-3 cells. The cells were incubated with the conjugates (2 nM) with (blocked) or without (non-blocked) pre-incubation with 200 nM of the same non-radiolabeled affibody conjugate. For all constructs, a significantly lower ($p < 0.0001$) signal was obtained in the blocked than in the non-blocked samples. The data are presented as the average ± 1 SD, $n = 3$.

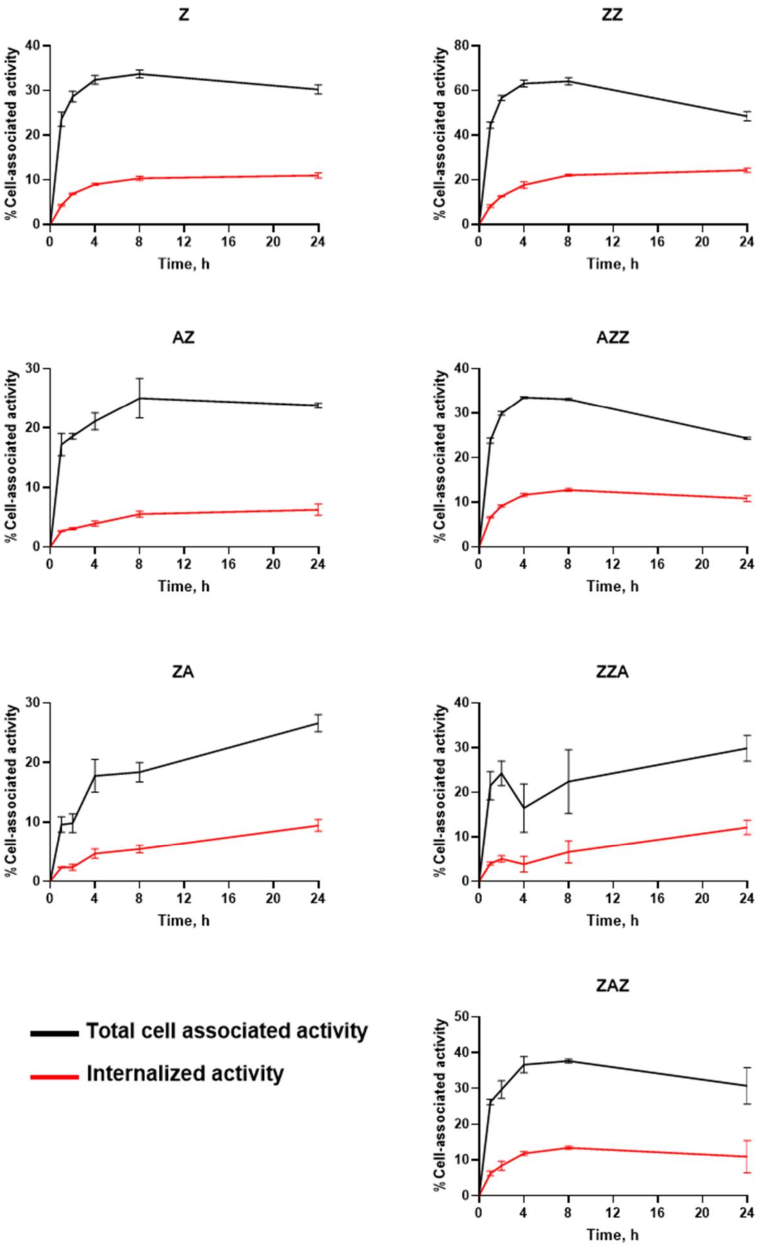

Figure S3. Cellular processing of the affibody constructs, where Z denotes $Z_{HER2:2891}$ and A denotes ABD_{035}, with architectures as described in Figure 1A. Processing of the affibody conjugates, labeled with technetium-99m, by HER2-positive SKOV-3 cells under continuous incubation, 2 nM, 37 °C. The membrane-bound activity was collected with a 0.2 M glycine buffer containing 4 M urea, pH 2.5, 5 min on ice, and the internalized activity was subsequently collected with 1 M NaOH. The data are presented as the average ± 1 SD, $n = 3$.

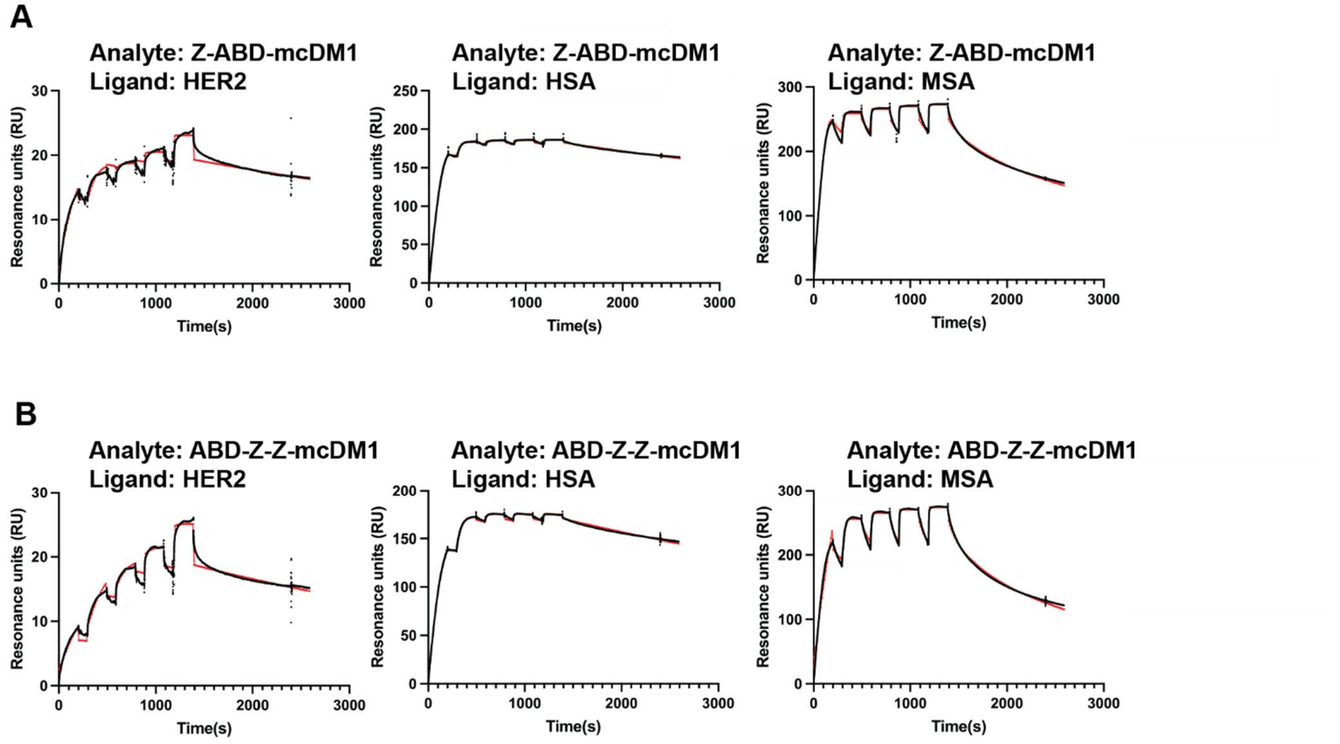

Figure S4. Measurement of the affinity of the drug conjugates. The interactions between HER2, HSA and MSA and the drug conjugates was investigated. Dilution series of the drug conjugates were sequentially injected in single-cycle analysis mode over flowcells with immobilized HER2, HSA or MSA. **(A)** The black curves correspond to representative sensorgrams recorded during the analysis with Z-ABD-mcDM1. **(B)** The black curves corresponds to representative sensorgrams recorded during the analysis with ZBD-Z-Z-mcDM1. The red curves in both panels correspond to the theoretical curves drawn based on the kinetic parameters of each interaction.

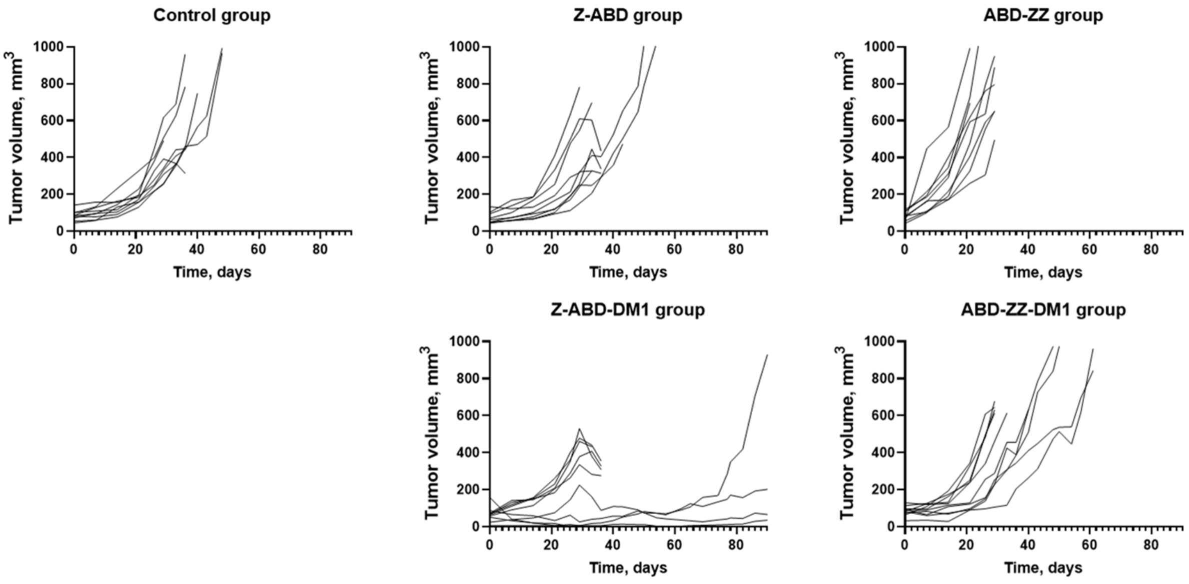

Figure S5. Tumor growth curves in individual mice. The animal were sacrificed when the tumors reached a critical volume of 1 cm^3, or tumor started ulcerating. Above each panel is indicated which drug conjugate (Z-ABD-DM1 or ABD-ZZ-DM1) or control construct (Z-ABD or ABD-ZZ) that was used for treatment. The control group did not receive any drug conjugate or control construct.

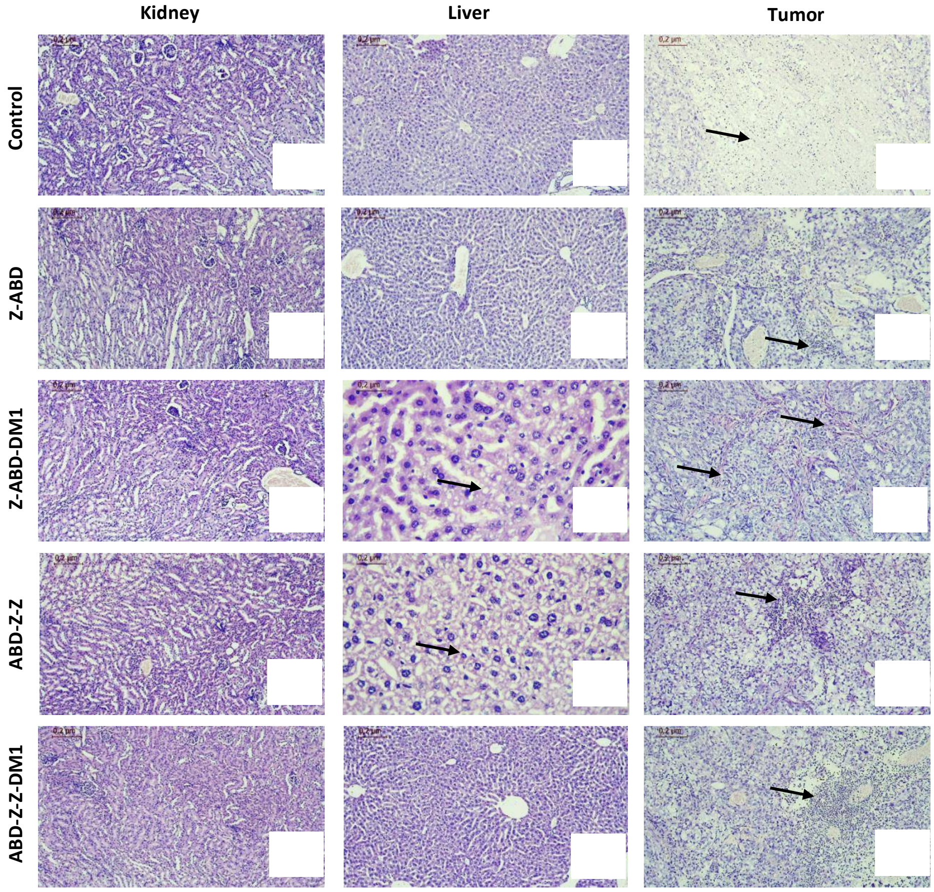

Figure S6. Results of the pathological examination. Histological sections of mices' kidneys, livers and tumors for control and exposed groups, stained by Hematoxylin-Eosin. The left panel (**A**–**F**) shows the absence of morphological alterations in the kidneys. The normal histological structure of the organ is preserved (original magnification ×10); (**G**,**H**,**K**)–liver without morphological lesion (original magnification ×10); (**I**,**J**)–mild fatty change (arrows) and a variation in nuclear feature of hepatocytes (original magnification ×40); (**L**): tumor necrosis in the central part of the image (original magnification ×10); (**M**,**O**,**P**)–the arrows indicate the foci of inflammation in the tumor (original magnification ×10); (**N**)–fibroblast bundles in the tumor (original magnification ×10).

Table S1. Biodistribution of the affibody-based fusion proteins labeled with technetium-99m at 4 and 24 h after iv injection in BALB/c nu/nu mice bearing HER2-positive SKOV-3 xenografts. The data are presented as % injected dose per g tissue (%ID/g), except for the data for the gastrointestinal tract and carcass, which are presented as % injected dose per sample.

	^{99m}Tc-Z$_{HER2}$	^{99m}Tc-ABD-Z$_{HER2}$	^{99m}Tc-Z$_{HER2}$-ABD	^{99m}Tc-Z$_{HER2}$-Z$_{HER2}$	^{99m}Tc-ABD-Z$_{HER2}$-Z$_{HER2}$	^{99m}Tc-Z$_{HER2}$-Z$_{HER2}$-ABD	^{99m}Tc-Z$_{HER2}$-ABD-Z$_{HER2}$
				**4h**			
Blood	1.5 ± 0.2	19.1 ± 0.5	21 ± 2	2.4 ± 0.1	15 ± 1	14 ± 2	15.1 ± 0.9
Salivary gland	0.5 ± 0.1	3.8 ± 0.8	3 ± 2	3.1 ± 1.0	4 ± 2	4 ± 1	4 ± 1
Lung	1.1 ± 0.2	8 ± 1	10 ± 1	2.73 ± 0.08	7 ± 3	7 ± 3	9 ± 1
Liver	3.5 ± 0.4	9.4 ± 0.6	11 ± 1	17 ± 1	22 ± 4	20 ± 4	20 ± 3
Spleen	1.6 ± 0.1	5.4 ± 0.6	7 ± 1	6.5 ± 0.8	9 ± 2	8 ± 2	9 ± 1
Stomach	0.37 ± 0.08	2.1 ± 0.3	2.6 ± 0.3	2.2 ± 0.7	2.9 ± 0.7	3.0 ± 0.5	3.0 ± 0.2
Kidney	202 ± 35	86 ± 12	66 ± 7	117 ± 10	69.1 ± 8.7	90 ± 18	62 ± 12
Tumor	12 ± 3	7 ± 1	6 ± 2	4.1 ± 0.2	4.2 ± 1.0	5 ± 1	6 ± 1
Muscle	0.13 ± 0.05	1.1 ± 0.2	1.1 ± 0.2	0.9 ± 0.3	1.0 ± 0.4	1.2 ± 0.2	1.08 ± 0.09
Bone	0.6 ± 0.3	2.1 ± 0.2	2.1 ± 0.2	1.5 ± 0.4	2.9 ± 0.6	2.4 ± 0.9	2.0 ± 0.5
Gastrointestinal tract	2.3 ± 0.3	4.0 ± 0.2	4.7 ± 0.5	7.0 ± 0.9	5.9 ± 0.6	5.0 ± 0.5	6 ± 1
Carcass	3.7 ± 0.3	27.8 ± 0.6	26.2 ± 0.3	17 ± 1	25 ± 1	23 ± 2	29 ± 1
				**24 h**			
Blood	0.46 ± 0.06	10 ± 1	9.8 ± 0.5	0.9 ± 0.1	4.7 ± 0.5	4.8 ± 0.8	5.6 ± 0.7
Salivary gland	0.3 ± 0.1	3 ± 1	3 ± 1	2.2 ± 0.2	2.2 ± 0.8	2.3 ± 1.0	2.7 ± 0.5
Lung	0.47 ± 0.09	5 ± 2	4 ± 2	1.1 ± 0.4	3.7 ± 0.3	3.4 ± 0.5	4.1 ± 0.2
Liver	2.5 ± 0.2	9.6 ± 0.9	10.5 ± 0.6	11 ± 2	11 ± 1	13 ± 3	12 ± 2
Spleen	0.8 ± 0.3	4.8 ± 0.9	6 ± 1	4 ± 1	5.9 ± 0.9	6 ± 3	7 ± 2
Stomach	0.26 ± 0.06	1.5 ± 0.1	1.4 ± 0.4	1.3 ± 0.4	1.3 ± 0.3	1.4 ± 0.2	1.7 ± 0.3
Kidney	129 ± 15	62 ± 11	47 ± 6	70 ± 11	39.6 ± 0.7	50 ± 10	40 ± 7
Tumor	10 ± 1	10.8 ± 0.9	13 ± 2	2.6 ± 0.5	6 ± 1	7 ± 2	10 ± 2
Muscle	0.09 ± 0.03	1.0 ± 0.2	0.9 ± 0.3	0.55 ± 0.07	0.7 ± 0.1	0.8 ± 0.1	1.0 ± 0.2
Bone	0.30 ± 0.07	1.66 ± 0.07	1.9 ± 0.1	1.1 ± 0.6	1.5 ± 0.5	1.7 ± 0.3	1.9 ± 0.3
Gastrointestinal tract	1.5 ± 0.5	4.4 ± 0.3	4.4 ± 0.4	3.2 ± 0.7	2.9 ± 0.3	3.9 ± 0.5	3.7 ± 0.4
Carcass	2.6 ± 0.4	23 ± 1	23.5 ± 0.5	10.2 ± 0.8	17 ± 1	17.1 ± 0.6	20 ± 2
